# Improving Obstetric Provider Congenital Cytomegalovirus Knowledge and Practices

**DOI:** 10.1155/2020/8875494

**Published:** 2020-11-08

**Authors:** Megan H. Pesch, Carter Anderson, Erika Mowers

**Affiliations:** ^1^Division of Developmental and Behavioral Pediatrics, Department of Pediatrics, 300 N. Ingalls Street, University of Michigan, Ann Arbor, USA; ^2^National CMV Foundation, Tampa, Florida, USA; ^3^Department of Obstetrics and Gynecology, St. Joseph Mercy Hospital System, 5301 McAuley Dr, Ypsilanti, Michigan 48197, USA

## Abstract

**Background:**

Congenital cytomegalovirus infection (cCMV) is the most common congenital infection. Antenatal education is proven to reduce cCMV risk. Little is known about obstetric provider knowledge and practice patterns around cCMV.

**Objectives:**

To evaluate obstetric provider knowledge and practice patterns regarding cCMV at baseline and again after a brief educational intervention.

**Methods:**

Obstetric providers (*N* = 53) at a US academic community hospital were invited to complete a survey regarding their knowledge and practice patterns around cCMV. Providers attended a brief presentation about cCMV and later were invited to repeat the same survey. Univariate statistics were calculated for baseline data, and prepost intervention comparison analyses were conducted.

**Results:**

Baseline cCMV knowledge was low at 49% (*M* = 17.54 out of a possible 36, SD 6.4), with most providers (51%) reporting never counseling pregnant patients about cCMV. Post intervention, overall cCMV knowledge increased to 80% (*M* = 29.33, SD 4.1, *p* < .001); provider intention to counsel about cCMV prevention increased to 100%.

**Conclusions:**

Obstetric provider knowledge about cCMV is low, which likely impacts their antenatal counseling. Educational initiatives to increase awareness about cCMV may increase antenatal education and thereby decrease the risk of cCMV.

## 1. Introduction

Congenital cytomegalovirus (cCMV) is the most common congenital infection in the United States (US), affecting one in every 150 live births [1]. Infants born with this preventable disease may have symptoms ranging from mild hearing loss to blindness, epilepsy, and spastic cerebral palsy [2]. A recent study found that only 9% of US women had ever heard of cCMV, highlighting the need for more antenatal education [1]. However, little is known about obstetric providers' own knowledge about cCMV, as well as their practice patterns around antenatal education.

Antenatal education about cCMV prevention has been shown to be effective in preventing seroconversion during pregnancy [3]. However, the American College of Obstetricians and Gynecologists (ACOG) does not recommend antenatal education, citing inconclusive evidence in their 2015 bulletin [4]. More recent studies have found straightforward educational initiatives to be both feasible and effective, spurring many to call for universal antenatal education on how women can lower their risk of cCMV [3]. For example, the risk of cCMV can be lowered by behavior change, specifically by avoiding contact with the bodily fluids of an individual who may be shedding the virus, most often young children, in the weeks before and during pregnancy. While several other countries recommend antenatal CMV education, this is not commonplace in the US, likely due in part to the lack of support by ACOG.

Women routinely rely and trust their obstetric providers for antenatal education. However, if provider knowledge about the risks of cCMV is low, women are less likely to receive counseling about preventative measures. A survey of US obstetricians in 2007 found that fewer than half (44%) of respondents had ever counseled their patients about cCMV prevention [5]. That survey also found low provider knowledge about cCMV prevention strategies, especially with regard to the important role children play in transmission of the virus [5]. In 2015, ACOG changed their recommendation to no longer support counseling pregnant women about cCMV [4]. Since that time there have been no surveys to gauge US obstetric provider knowledge or practice patterns around antenatal cCMV counseling. Furthermore, no studies to date have evaluated the impact of provider targeted educational interventions on both cCMV knowledge and practice patterns. Increasing obstetric provider knowledge about cCMV is an essential step to influence their antenatal education practices.

Therefore, the objectives of this study were two-fold: [1] to assess baseline obstetric provider overall cCMV knowledge, knowledge perception, and practice patterns; and [2] to assess change in provider cCMV knowledge, knowledge perception, and intended practice patterns after a brief educational intervention.

## 2. Methods

### 2.1. Study Overview

This study involved a pre-post administration of a survey designed to assess obstetrical provider knowledge and practice patterns concerning cCMV. The intervention, described below, was a brief 20-minute Powerpoint presentation on cCMV. Baseline and prepost comparison statistics were calculated.

### 2.2. Participants and Recruitment

Obstetric providers at an academic community hospital were invited in January 2020 to complete an online survey about their knowledge and practice patterns concerning cCMV. Inclusion criteria included that participants were a physician, midwife, or advanced care practitioner in the institution's Department of Obstetrics and Gynecology. Ten days prior to the planned intervention, an email was sent out with a SurveyMonkey™ link, inviting participants to complete the survey. Two additional reminder emails were sent out to the providers prior to the presentation, which was scheduled for Grand Rounds. Two days after the intervention, the same survey was sent out to the group with some question modifications about intent for practice change. Participants were not compensated for their participation in this study. This study was approved as exempt by the Institutional Research Board, as such informed consent forms were not collected.

### 2.3. Intervention

A 20-minute Powerpoint style presentation covering the prevalence, diagnosis, outcomes, treatment options of cCMV, and evidence supporting antenatal education was given at the department grand rounds meeting by two study team members. This presentation is available from the authors upon request. A brief question and answer period was included at the end of the intervention, which lasted 5 minutes.

### 2.4. Measures

A 22 item questionnaire (available from the authors upon request) was designed to evaluate cCMV knowledge and practice patterns, based off prior work [6]. The questionnaire consisted of an overall cCMV knowledge subscale (11 multiple-choice questions, for a total possible score of 36) and three additional subscales: perceived knowledge (8 items, 5-point ordinal Likert scale ranging from 1 = poor to 5 = excellent), counseling on prevention (2 items, 5-point ordinal Likert scale ranging from 1 = never to 5 = 76-100% of the time), and testing frequency (1 item, 5-point ordinal Likert scale ranging from 1 = never to 5 = 76-100% of the time). The survey was repeated after the presentation, with alterations to the subscale questions to indicate future practice intent. Providers reported their role (i.e., faculty, resident, and midwife), and the number of years they had been in practice.

### 2.5. Statistical Analyses

Analyses were done with the JMP software (JMP®, Version 14.0. SAS Institute Inc., Cary, NC, 2020). Baseline descriptive data were computed. Analysis of variance (ANOVA) was done to compare attending, resident, and midwife/APP roles for the initial knowledge test scores. The midwives and one advanced practice practitioner (APP) respondent were grouped for this comparison. ANOVA was used to compare initial knowledge scores for years of experience groupings for the 34 attending physician providers. For the second objective, cCMV knowledge scores for the 24 respondents completing pre- and posttests were compared using a paired *t*-test. Likert-type data of perceived knowledge, counseling, and testing were analyzed utilizing a Wilcoxon signed-rank test for paired data, because the ordinal data are not from a continuous normal distribution.

## 3. Results

Characteristics of the sample are provided in Tables 1 and 2. A total of 53 respondents completed the initial pre-intervention survey, 30 completed the post-intervention survey, and 24 participants responded to both.

The results of the baseline data are presented in Table 1. In brief, the mean Overall cCMV Knowledge score for the sample was 17.5 points out of a possible 36 (SE = 6.43), which is just less than 50% correct. Faculty and Resident Overall cCMV Knowledge scores were statistically higher than those of Midwives and APP (Mean = 11 points, SD = 4.7, *p* = .012). As a whole, participants rated themselves as having fair perceived knowledge of cCMV (*M* = 2.13, SD = 0.83). In terms of counseling practices, the majority of respondents reported that they never (51%, *n* = 26) or rarely (47%, *n* = 24) counseled expectant mothers about the risks and prevention of cCMV. Most respondents reported that they never (25%, *n* = 13) or rarely (73%, *n* = 37) tested expectant mothers with cold-like symptoms for CMV.

New faculty (0-5 years in practice) scored the highest in Overall cCMV Knowledge (Mean = 21.5, SE = 2.5), but there were no statistical differences between the faculty groups based on numbers of years in practice (see Table 2).

After the educational intervention, scores in all domains increased for those completing both surveys (Table 3). As a whole, Overall cCMV Knowledge increased to a mean score of 81.5% correct (*M* = 29.33 points, SD = 4.10). For the individuals who completed both surveys (*n* = 24), there was an improvement in their Overall cCMV Knowledge of 9.67 points (SE = 0.97) or 26.9% more answers correct (*p* < .0001). Postintervention, counseling on prevention intention increased to the “often to always” range (from “never to rarely” at baseline) (*p* < .001). No respondents stated they will never counsel, 26.67% stated they will counsel 1-25% of the time, 13.33% will counsel 26-50% of the time, 23.33% will counsel 51-75% of the time, and 36.67% will counsel 76-100% of the time. Following the educational session, 100% of respondents reported intent to increase antenatal cCMV counseling, with the mean frequency of testing intention increased to 2.4, on a 3-point scale (1 = never, 2 = high-risk patients, 3 = all patients), (range 2-3, *p* < .001)

## 4. Discussion

In this study, obstetric providers were found to have low baseline knowledge about cCMV and infrequent practices around counseling and screening their patients. Findings from the study support prior literature on the knowledge and practice patterns of obstetric providersaround cCMV [5]. More importantly, this study demonstrates that a short educational intervention can increase provider knowledge and intentions to counsel and screen for cCMV in their practices. The intervention delivered in this study was straightforward and could be easily adopted by other health systems and practices.

Many argue that antenatal education about cCMV prevention should be reflexive and a part of routine antenatal care [1, 2, 7, 8]. While performing such additional education during an already busy clinical visit will take more time, some content that antenatal cCMV education should take precedence over many of the other non-evidence-based and low-yield topics routinely covered during these visits (e.g., avoidance of artificial sweeteners, caffeine, sushi, and hair dye) [9]. Perhaps the primary reason for low antenatal counseling and testing is low provider awareness about cCMV. Our study demonstrates that as provider knowledge increased, so did their intention to counsel and screen for cCMV. This trend is likely applicable to many obstetric providers, as there is little emphasis on cCMV in obstetrical training [10]. While it is the prerogative of each practitioner whether or not to counsel their patients about cCMV, it is the responsibility of national and international organizations such as the American Academy of Pediatrics, ACOG, and the World Health Organization to increase provider and public awareness about this common infection. Increasing provider and public knowledge about the dangers of cCMV is critical to the success of vaccines currently in phase 2 and phase 3 trials in the US. Wide acceptance of a CMV vaccine could drastically reduce the incidence of congenital cases and resultant hearing loss/neurodevelopmental disabilities. Future studies are needed to examine how to best increase provider knowledge about cCMV in different practice settings, as well as how this increased knowledge translates into measurable practice change and seroconversion in their patients.

This study is not without limitations. This work is the result of a single educational session with self-report pre-post surveys of knowledge and practice patterns but did not measure actual practice change in providers. This study also did not measure the persistence of increased cCMV knowledge over time. This work is also limited by the low sample size and should be reproduced in larger samples. Results may not be generalizable to other populations.

## 5. Conclusions

This study found a low baseline level of cCMV knowledge and infrequent antenatal counseling and screening practices among obstetric providers. A brief educational intervention increased overall provider cCMV knowledge and intent to provide antenatal counseling and screening. Increased antenatal education around cCMV prevention may decrease the incidence of cCMV. Obstetric providers may benefit from increased opportunities to learn about cCMV during their training and throughout their careers.

## Figures and Tables

**Table 1:** Participant characteristics and scores at baseline.

Provider role		Overall cCMV knowledge		Perceived knowledge	Counseling on prevention	Testing frequency
	*N* (%)	Mean (SD)	Group^ϯ^	Mean (SD)	Mean (SD)	Mean (SD)
All respondents	53 (100%)	17.55 (6.43)		2.13 (0.83)	1.53 (0.61)	1.80 (0.63)
Faculty physician	34 (64%)	18.12 (6.10)	A	2.12 (0.91)	1.52 (0.51)	1.91 (0.69)
Resident physician	12 (23%)	19.67 (6.30)	A	2.33 (0.65)	1.36 (0.50)	1.58 (0.51)
Midwife or AAP	7 (13%)	11.14 (4.74)	B	1.86 (0.69)	1.86 (1.07)	1.71 (0.49)
Significance probability		0.012				

^ϯ^Groups not connected by the same letter are significantly different at *p* < 0.05.

**Table 2:** Baseline respondent years in practice and faculty physician cCMV knowledge scores.

	All respondents	Overall cCMV knowledge for the 34 faculty physicians			
Years in practice (years)	*N* (%)	*N* (%)	Mean	Group^ϯ^	Standard error of mean
0-5	22 (41.9%)	6 (20.9%)	21.5	A	2.5
6-10	7 (12.6%)	7 (19.0%)	16.7	A	2.4
11-20	11 (21.3%)	10 (27.9%)	17.2	A	2.0
21-30	9 (16.2%)	8 (22.6%)	17.4	A	2.2
31+	4 (8.0%)	3 (9.6%)	19.7	A	3.6
Significance probability				0.625	

^ϯ^Groups not connected by the same letter are significantly different at *p* < 0.05.

**Table 3:** Differences of final minus initial cCMV knowledge and final minus initial Likert-type responses for the 24 dual survey completers.

	Overall cCMV knowledge change	Perceived knowledge change	Counseling on prevention change	Testing frequency change
Mean difference	9.67	1.25	2.25	0.71
Standard error of mean	0.97	0.19	0.26	0.20
N	24	24	24	24
Prob. > |t|	<0.0001^ϯ^	<0.0001^ϯϯ^	<0.0001^ϯϯ^	0.0002^ϯϯ^

^ϯ^Tested with paired *t*-test using Student's *t* distribution. ^ϯϯ^Tested with Wilcoxon signed-rank test for paired data.

## Data Availability

Data is available from the authors upon request. Please contact pesch@umich.edu.
